# Regulation of glutamate transport and neuroinflammation in a term newborn rat model of hypoxic–ischaemic brain injury

**DOI:** 10.1177/23982128221097568

**Published:** 2022-05-20

**Authors:** Silvia Pregnolato, Hemmen Sabir, Karen Luyt, Kira DA Rienecker, Anthony R Isles, Elavazhagan Chakkarapani

**Affiliations:** 1 Department of Neonatal Neurology, Bristol Medical School, University of Bristol, Bristol, UK; 2 Department of Neonatology and Pediatric Intensive Care, Children’s Hospital, University of Bonn, Bonn, Germany; 3 Department of Pediatrics I/Neonatology, University Hospital Essen, University Duisburg Essen, Essen, Germany; 4 Department of Physical Therapy and Rehabilitation Science, University of California San Francisco, San Francisco, CA, USA; 5 Behavioural Genetics Group, MRC Centre for Neuropsychiatric Genetics and Genomics, School of Medicine, Cardiff University, Cardiff, UK

**Keywords:** Hypoxic–ischemic encephalopathy, neuroinflammation, glutamate, hypoxia–ischaemia, cerebral ischaemia, newborn, neonatal, brain

## Abstract

In the newborn brain, moderate-severe hypoxia–ischaemia induces glutamate excitotoxicity and inflammation, possibly via dysregulation of candidate astrocytic glutamate transporter (*Glt1*) and pro-inflammatory cytokines (e.g. *Tnfα*, *Il1β*, *Il6*). Epigenetic mechanisms may mediate dysregulation. Hypotheses: (1) hypoxia–ischaemia dysregulates mRNA expression of these candidate genes; (2) expression changes in *Glt1* are mediated by DNA methylation changes; and (3) methylation values in brain and blood are correlated. Seven-day-old rat pups (*n* = 42) were assigned to nine groups based on treatment (for each timepoint: naïve (*n* = 3), sham (*n* = 3), hypoxia–ischaemia (*n* = 8) and timepoint for tissue collection (6, 12 and 24 h post-hypoxia). Moderate hypoxic–ischemic brain injury was induced via ligation of the left common carotid artery followed by 100 min hypoxia (8% O_2_, 36°C). mRNA was quantified in cortex and hippocampus for the candidate genes, myelin (*Mbp*), astrocytic (*Gfap*) and neuronal (*Map2*) markers (qPCR). DNA methylation was measured for *Glt1* in cortex and blood (bisulphite pyrosequencing). Hypoxia–ischaemia induced pro-inflammatory cytokine upregulation in both brain regions at 6 h. This was accompanied by gene expression changes potentially indicating onset of astrogliosis and myelin injury. There were no significant changes in expression or promoter DNA methylation of *Glt1*. This pilot study supports accumulating evidence that hypoxia–ischaemia causes neuroinflammation in the newborn brain and prioritises further expression and DNA methylation analyses focusing on this pathway. Epigenetic blood biomarkers may facilitate identification of high-risk newborns at birth, maximising chances of neuroprotective interventions.

## Introduction

Hypoxic–ischemic encephalopathy (HIE) is a brain dysfunction due to blood/oxygen deprivation around the time of birth, with prevalence of 2–3/1000 live births or higher wherever availability of advanced perinatal care is limited (Gale et al., 2018; Jacobs et al., 2013; United Nations Inter-agency Group for Child Mortality Estimation (UN-IGME), 2019). Therapeutic hypothermia is the only neuroprotective treatment for term newborns with moderate to severe HIE (Azzopardi et al., 2009; Gluckman et al., 2005; Jacobs et al., 2011, 2013; Shankaran et al., 2005; Simbruner et al., 2010; Zhou et al., 2010). Despite cooling, 11% of cooled newborns still die, 20% develop cerebral palsy (CP) with moderate to severe cognitive impairment (Shankaran et al., 2017), and 25%–60% develop milder cognitive impairment and behavioural problems (Lee-Kelland et al., 2020; Schreglmann et al., 2020).

The clinician must make rapid decisions at birth to ensure intervention within the therapeutic window preceding secondary brain injury (Groenendaal, 2019; Thoresen et al., 2013). Serum levels of proteins dysregulated during the injury process are being investigated as biomarkers to promote early identification of newborns who may benefit from additional neuroprotective interventions currently being developed (Douglas-Escobar and Weiss, 2012; Graham et al., 2018; Lv et al., 2015). A category of molecular biomarkers, which awaits exploration in the context of HIE is represented by epigenetic biomarkers. DNA methylation is key for brain function and development, is responsive to early life environmental stresses, and its dysregulation and association with gene silencing has been observed in a range of brain disorders (Greenberg and Bourc’his, 2019; Jin and Liu, 2018; Petronis, 2010). Changes in DNA methylation can act as a code of previous experience that results in a differential response to a future exposure (Isles, 2015). The relative stability and ease of measurement are attractive properties, and cord blood DNA methylation is currently being assessed in relation to a range of early life stresses leading to neurodevelopmental impairment (Hodyl et al., 2016).

Following hypoxia–ischaemia, failure of astrocytic glutamate transporters to clear glutamate from the synaptic cleft contributes to postsynaptic glutamate receptor overactivation. The resulting glutamate excitotoxicity ultimately leads to cell damage or death within hours (Benveniste et al., 1984; Choi, 1992; Danbolt, 2001; Olney, 1969; Thornton et al., 2012; Vandenberg and Ryan, 2013). *Glt1* (human orthologue: *SLC1A2* or *EAAT2*) is the main glutamate transporter in the mammalian forebrain, being responsible of up to 95% glutamate transport (Danbolt, 2001; Haugeto et al., 1996). It is dysregulated in several adult excitotoxic disorders (Fontana, 2015; Karki et al., 2015; Takahashi et al., 2015) and preterm white matter injury (DeSilva et al., 2008; Pregnolato et al., 2019), whereas it has not been comprehensively explored in the context of HIE. Elevated glutamate levels are found in basal ganglia and cerebrospinal fluid of newborns with HIE, suggesting impaired transport (Gücüyener et al., 1999; Hagberg et al., 1993; Pu et al., 2000; Riikonen et al., 1992). Candidacy is supported by evidence of dysregulation following hypoxia–ischaemia in the term-equivalent piglet (Martin et al., 1997; Pow et al., 2004; Reye et al., 2002).

In this pilot study, we focused on transcriptional and epigenetic regulation of *Glt1*. We further expanded the research question by investigating gene expression changes related to neuroinflammation. Emerging evidence indicates that hypoxia–ischaemia causes an inflammatory response in the brain, with activation of microglia by the excitotoxically injured neurons and infiltration of peripheral immune cells via the disrupted blood–brain barrier (Algra et al., 2013; Hagberg et al., 2015; Winerdal et al., 2012; Yong and Marks, 2010). Levels of pro-inflammatory cytokines tumour necrosis factor alpha (TNFα), interleukin (IL)1β and IL6 have been shown to predict neurodevelopmental impairment in at-risk newborns (Magalhães et al., 2019; Nist and Pickler, 2019).

Using the Vannucci rat model, we hypothesised that hypoxia–ischaemia alters *Glt1, Tnf*α, *Il1*β and *Il6* transcription in the first 24 h in two of the most vulnerable brain regions, cortex and hippocampus (Towfighi et al., 1991; Vannucci and Vannucci, 2005). In addition, *Glt1* promoter DNA methylation was assessed in the cortex and peripheral blood, to evaluate translational potential as a clinical biomarker for HIE. We show that hypoxia–ischaemia increases transcription of key pro-inflammatory cytokines but does not affect regulation of the glutamate transporter in the newborn rat brain.

## Methods

### Animals and experimental procedure

All experiments were performed in accordance with the Animal Research: Reporting of in vivo Experiments (ARRIVE) guidelines with government approval by the State Agency of Nature, Environment and Consumer Protection North Rhine–Westphalia, Germany. Between experiments, pups were kept with their dams at the central animal facility at University Hospital Essen, Germany, with a 12 h:12 h dark:light cycle at 21°C room temperature and access to food and water *ad libitum*. A total of 43 7-day-old (P7) Wistar rat pups from four litters were used in this study, with one rat serving as a ‘sentinel’ for temperature monitoring and not included in the final sample (*n* = 42: *n* = 24 male, *n* = 18 female). Temperature of the sentinel rat was measured via a rectal probe (IT-21, Physitemp Instruments, Clifton, NJ, United States). Rectal temperature, which correlates with brain temperature within 0.1°C (Thoresen et al., 1996), was continuously maintained within ± 0.2°C of the target nesting temperature (38.5°C) and target treatment temperature (36°C) using a servo-controlled water-filled mat (CritiCool, MTRE, Yavne, Israel). This treatment temperature has been previously shown to contribute to moderate brain injury in this model, with approximately 40% area loss in left (injured) versus right (uninjured) hemisphere (Sabir et al., 2012; Thoresen et al., 1996; Wood et al., 2016a).

**Figure 1.**
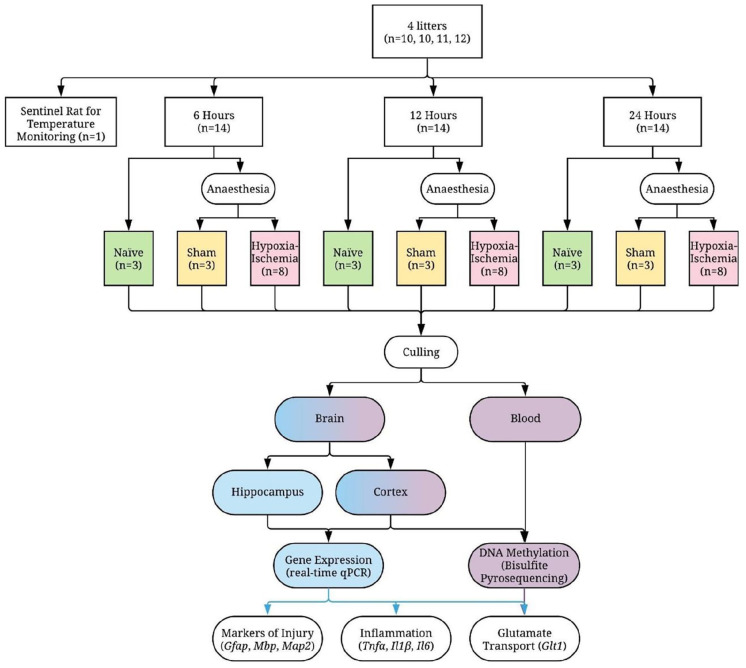
Experimental design.

The 42 pups were assigned to three experimental groups (naïve, surgical sham and hypoxia–ischaemia) at three timepoints (6, 12 and 24 h post-hypoxia), ensuring balanced representation of litter, sex and birth weight in each group to minimise confounding (Figure 1). The a priori unbalanced design (*n* = 14 for each of the three timepoints: *n* = 3 for naïve, *n* = 3 for surgical sham and *n* = 8 for hypoxia–ischaemia) was based on the known wide distribution of injury in this model (Sabir et al., 2012; Thoresen et al., 1996; Wood et al., 2016a). Twenty-four pups were male (57%), with the naïve and sham groups at each timepoint including one or two males out of three pups, and the hypoxia–ischaemia groups including four or five males out of eight pups. Both sham and hypoxia–ischaemia groups underwent isoflurane anaesthesia (3% isoflurane in a 2:1 gas mixture of NO_2_/O_2_, gas flow of 1.2 l). Hypoxia–ischaemia was induced by left carotid artery ligation followed by exposure to hypoxia. The average time for anaesthesia and ligation was 7 min and ligations were performed by a single operator (HS). After 30 min post-surgery recovery with the dam, pups were exposed to 8% oxygen for 100 min at 36°C in a custom-designed chamber (Hobbs et al., 2008; Wood et al., 2016a).

Animals were sacrificed at median times of 6, 12 and 24 h post-hypoxia by rapid decapitation using surgical scissors. Peripheral blood was collected immediately after decapitation using K_2_EDTA-anticoagulated tubes. Cortex and hippocampus were isolated from a single 2.5 mm slice collected using a standard rat brain matrix (Zivic Instruments).

### Gene expression analysis

Gene expression was assessed via reverse transcriptase-quantitative polymerase chain reaction (RT-qPCR) in the cortex and hippocampus for the following genes: the glutamate transporter (*Glt1*), three pro-inflammatory cytokines (*Tnfα*, *Il1β* and *Il6*) and three established markers chosen to monitor early transcriptional responses potentially signalling brain injury in the absence of histological and survival data, that is, a marker of astrogliosis (*Gfap*), a marker of neuronal injury (*Map2*) and a marker of myelin injury (*Mbp*).

DNA and mRNA were extracted from each brain sample using TRIzol (Thermo Fisher Scientific), ensuring they originated from the same cell population. mRNA was cleaned using a column-kit (RNeasy MinElute, Qiagen) and treated with DNase (Ambion). One microgram of mRNA per sample was reverse-transcribed to cDNA (RNA to cDNA EcoDry™ Premix (Double Primed) kit, Takara) and amplified in triplicate via qPCR using the intercalating SYBR Green fluorescent dye (Bioline). PCR amplification was carried out in 100-well rings on the Rotor-Gene Q cycler (Qiagen). Amplification protocol and primer sequences are shown in the Supplementary Material (Supplementary Section 1.1.1). Primers were designed to span intron–exon boundaries universal to all known isoforms for each gene, ensuring selective amplification of mRNA over genomic DNA. Fluorescence was normalised to the geometric mean of two housekeeping genes (*Hprt* and *B2m*) to account for non-biological variation in gene expression (Supplementary Section 1.2.1.1 in the Supplementary Material). Gene expression data were analysed with the 2^−ΔΔCt^ method (Livak and Schmittgen, 2001) to obtain Δ*Ct* values for statistical analyses and plots (i.e. normalised gene expression on the log scale) and 2^ΔΔCt^ values for plots (i.e. fold change relative to the timepoint-matched naïve group).

### DNA methylation analysis

**Figure 2.**
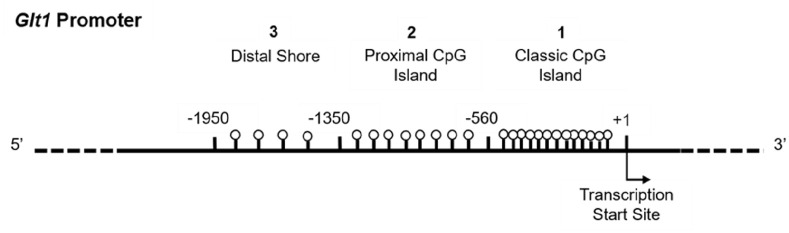
Diagram of the candidate promoter regions in *Glt1* explored in the DNA methylation analysis.

DNA methylation was assessed via bisulphite conversion followed by pyrosequencing in cortex and blood for three candidate regions in the glutamate transporter (Figure 2). These included a region in the classic CpG island and an upstream shore region, showing differential methylation and expression patterns in the rat brain (Perisic et al., 2010, 2012; Zschocke et al., 2005). A proximal region was also included that is homologous to a region differentially methylated in saliva of very preterm versus healthy term newborns in a hypothesis-free epigenome-wide association study (EWAS) (Sparrow et al., 2016).

Analysis of *Peg3* was included as a positive control. As an imprinted gene, *Peg3* should show approximately 50% methylation in all cells, providing an internal control for successful bisulphite conversion and pyrosequencing. Given the lack of *Peg3* studies in rat in the wider literature, primers were designed to target a small region homologous to the mouse imprinted differentially methylated region (DMR) (Zhan et al., 2017). Pyrosequencing technology can robustly sequence small regions of the genome (up to 60–80 bp), and the number of CpG sites varies by region depending on CpG density (*Glt1* CpG island: seven CpGs; *Glt1* proximal promoter: three CpGs; *Glt1* distal shore: two CpGs and *Peg3*: five CpGs).

DNA from both brain and blood was cleaned with a column-kit (DNeasy Blood & Tissue kit, Qiagen). A total of 200-500 ng DNA (quantified via Qubit fluorometer, Thermo Fisher Scientific) was subjected to bisulphite conversion (EZ DNA Methylation-Gold kit, Zymo Research), which converts unmethylated but not methylated cytosines into uracils. Bisulphite-converted DNA for each candidate region was amplified and biotin-tagged via a two-step PCR on a standard thermal cycler (Bio-Rad) (Supplementary Section 1.1.2 in the Supplementary Material). The two-step PCR approach increases the amount of amplified PCR products available for analysis from the source DNA, while also allowing use of a single biotinylated primer for all candidate regions assessed. This is achieved by incorporating an arbitrary common primer sequence in the region-specific amplified products in the first PCR, which is recognised and amplified by a biotinylated common primer in the second PCR (Royo et al., 2007; Woodfine et al., 2011). Primers for both PCR amplification and pyrosequencing were designed using the Pyromark Assay Design 2.0 software (Qiagen) and are reported in the Supplementary Material (Supplementary Section 1.1.2). Whenever avoiding a CpG site in a primer sequence was not possible, a mix of primers containing all four bases at the CpG site was used, since this method has been previously shown to produce the least bias at similar melting temperatures (Candiloro et al., 2017). Biotin-tagged strands were subsequently isolated via streptavidin beads and sequenced by pyrosequencing on the Pyromark Q96 (Qiagen) in triplicates. The per-CpG DNA methylation data were averaged to obtain a single methylation value for each promoter region. This is based on evidence that there is strong correlation between neighbouring CpG sites within 1–2 kb regions in the same cell types (Ball et al., 2009; Bell et al., 2011; Bibikova et al., 2011; Eckhardt et al., 2006; Shoemaker et al., 2010), therefore, reducing data to regions can increase power and reduce noise (Hogg et al., 2014).

### Statistical analysis

The exposure in this study is experimental group (naïve, sham and hypoxia–ischaemia) and the outcomes are normalised gene expression on the log scale (glutamate transporter and pro-inflammatory cytokines; cortex and hippocampus) and percentage DNA methylation at each candidate promoter region (glutamate transporter; cortex and blood). Group effects were tested separately for each of the three timepoints (6, 12 and 24 h) with Kruskal–Wallis tests, given the non-normal distribution of injury in this model (Wood et al., 2020). Post hoc analyses between pairs of groups were performed with the Dunn’s test.

Secondary analyses of suggestive value were planned to address the issue of limited power. Specifically, if no differences were detected, naïve and sham groups were collapsed into a single larger control group (*n* = 6 per timepoint) and differences with the hypoxia–ischaemia group tested with a Mann–Whitney test, separately for each timepoint.

Overall, three tests (one for each timepoint) were performed for each gene in each of the two brain regions. *p*-values are reported uncorrected for multiple comparisons. The Benjamini–Hochberg false discovery rate (FDR) was applied as an alternative strategy, since it is less penalising in terms of power by setting an acceptable level of false discoveries in this pilot study, and does not assume independency of tests for genes expected to be co-regulated (Benjamini and Hochberg, 1995). This value was set to 10% for both gene expression and DNA methylation data, and FDR calculations performed separately for each timepoint in each region.

Calculations from *Ct* values to 2^ΔΔCt^ values were performed using Excel (Microsoft Corp, version for Microsoft 365). All statistical analyses were carried out using Stata 14 (Stata Corp, TX, USA).

## Results

### Hypoxia–ischaemia induces gene expression changes suggesting astrogliosis and myelin injury in both cortex and hippocampus

Complete results and plots for the markers of injury are reported in the Supplementary Material (Supplementary Section 1.2.1.2). Briefly, there was significant evidence that hypoxia–ischaemia induced upregulation of *Gfap*, a marker of astrogliosis, at 6 h in both cortex (*p* = 0.021) and hippocampus (*p* = 0.018). In the cortex, *Gfap* was additionally upregulated at 12 h (*p* = 0.016), with weaker evidence in the hippocampus (*p* = 0.095). Evidence was not significant at 24 h in either region (*p* = 0.151), however, distribution appeared to be bimodal, with a non-significant trend for higher expression in the later phase (24 h) compared to the earlier phases (6 and 12 h) for a subset of these animals, especially in the cortex. Evidence strengthened in secondary analyses merging naïve and sham groups (cortex at 24 h: *p* = 0.053).

There was also significant evidence of a loss of *Mbp*, a marker of myelin injury, following hypoxia–ischaemia in the cortex at 6 h (*p* = 0.031) and 12 h (*p* = 0.041), with weaker evidence at 24 h (*p* = 0.064). This group effect was not observed in the hippocampus (6 h: *p* = 0.860; 12 h: *p* = 0.135 and 24 h: *p* = 0.110). However, variability in the naïve group was particularly high for hippocampal *Mbp*, and in the secondary analyses merging naïve and sham groups, a non-significant trend for lower expression after hypoxia–ischaemia was observed at 24 h (*p* = 0.071).

There was no evidence that hypoxia–ischaemia altered regulation of neuronal marker *Map2* in either cortex (6 h: *p* = 0.995; 12 h: *p* = 0.254 and 24 h: *p* = 0.257) or hippocampus (6 h: *p* = 0.576; 12 h: *p* = 0.658 and 24 h: *p* = 0.399). Overall, group effects at 6 h for *Gfap* in both brain regions and *Mbp* in the cortex surpassed Benjamini–Hochberg FDR (Supplementary Section 1.2.1.4 in the Supplementary Material).

### Hypoxia–ischaemia does not significantly alter regulation of the glutamate transporter in bulk tissue from cortex and hippocampus

**Figure 3.**
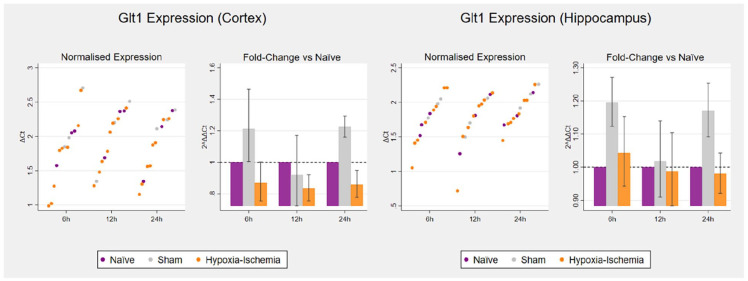
*Glt1* expression following hypoxia–ischaemia in the cortex (left) and hippocampus (right).

There were no significant changes in *Glt1* expression following hypoxia–ischaemia in either cortex (6 h: *p* = 0.386; 12 h: *p* = 0.562 and 24 h: *p* = 0.180) or hippocampus (6 h: *p* = 0.443; 12 h: *p* = 0.995 and 24 h: *p* = 0.262) (Table 1, Figure 3).

**Table 1.** *Glt1* expression: primary and secondary analyses.

Tissue	Analyses	Timepoint (h)	*p*
Cortex	Primary (*N* versus *S* versus *HI*)	6	0.386
		12	0.562
		24	0.180
	Secondary (*N* versus *S*)	6	0.827
		12	0.827
		24	0.513
	Secondary (*N/S* versus *HI*)	6	0.197
		12	0.302
		24	0.093
Hippocampus	Primary (*N* versus *S* versus *HI*)	6	0.433
		12	0.995
		24	0.262
	Secondary (*N* versus *S*)	6	0.127
		12	0.827
		24	0.275
	Secondary (*N/S* versus *HI*)	6	0.796
		12	> 0.999
		24	0.302

For each of the three timepoints for tissue collection (6, 12 and 24 h post-hypoxia): naïve (*n* = 3), sham (*n* = 3) and hypoxia–ischaemia (*n* = 8). N: naïve; S: sham and HI: hypoxia–ischaemia.

The scatterplots on the left reproduce untransformed Δ*Ct* values on the logarithmic scale. The bar charts on the right represent 2^ΔΔCt^ values, that is, the fold change on the linear scale in relation to respective age-matched controls, with potentiated means, potentiated asymmetric standard error bars and age-matched controls set as *y* = 1. For each of the three timepoints for tissue collection (6, 12 and 24 h post-hypoxia): naïve (*n* = 3), sham (*n* = 3) and hypoxia–ischaemia (*n* = 8).

**Figure 4.**
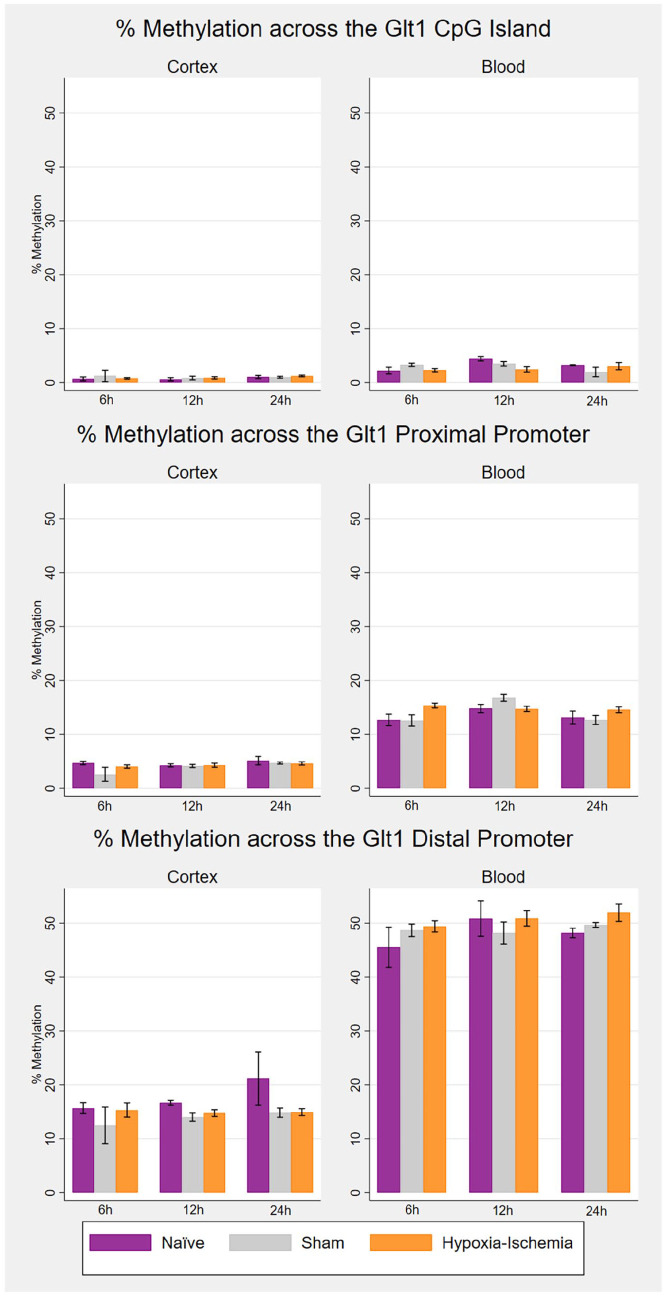
*Glt1* promoter DNA methylation following HI in cortex and blood.

The mean percentage methylation across the *Peg3* DMR was ~50% in both cortex and blood (Supplementary Section 1.2.2.1 in the Supplementary Material). With regard to the glutamate transporter, the CpG island was unmethylated (< 5%) in both cortex and blood across all groups and time points. The proximal promoter was unmethylated in the cortex (< 5%) and hypomethylated in blood (< 20%), and the *Glt1* distal shore was hypomethylated in the cortex (< 20%) and around 50% methylated in blood across all groups and time points (Figure 4). The complete methylation analyses for *Glt1*, including raw per-CpG methylation data and secondary analyses, are reported in the Supplementary Material (Supplementary Section 1.2.2.2). Overall, there were no significant methylation differences at any of the *Glt1* promoter regions in the cortex. In blood, there was some evidence of a group effect in the CpG island at 12 h (*p* = 0.040) and in the proximal promoter at 6 h (*p* = 0.035) (Table 2). According to post hoc analyses, the hypoxia–ischaemia group showed slightly lower methylation compared to the naïve group in the CpG island at 12 h (*p* = 0.006) and slightly higher methylation compared to both naïve (*p* = 0.026) and sham (*p* = 0.015) groups in the proximal promoter at 6 h. However, these differences were less than 5% in both cases, were not maintained through subsequent timepoints and were not correlated with expression changes in the cortex. Overall, no group effects in the DNA methylation analyses surpassed Benjamini–Hochberg FDR (Supplementary Section 1.2.2.3 in the Supplementary Material).

**Table 2.** *Glt1* promoter DNA methylation: primary analyses.

Tissue	*Glt1* promoter region	Timepoint (h)	*p*	Post hoc Dunn’s *p*
Cortex	CpG island	6	0.910	
		12	0.582	
		24	0.640	
	Proximal promoter	6	0.221	
		12	0.995	
		24	0.910	
	Distal shore	6	0.708	
		12	0.101	
		24	0.278	
Blood	CpG island	6	0.304	
		12	0.040	N ≠ HI (*p* = 0.006)
		24	0.285	
	Proximal promoter	6	0.035	N (*p* = 0.026) and S (*p* = 0.015) ≠ HI
		12	0.169	
		24	0.230	
	Distal shore	6	0.732	
		12	0.545	
		24	0.078	N ≠ HI (*p* = 0.016)

For each of the three timepoints for tissue collection (6, 12 and 24 h post-hypoxia): naïve (*n* = 3), sham (*n* = 3) and hypoxia–ischaemia (*n* = 8). N: naïve; S: sham and HI: hypoxia–ischaemia.

The bar charts represent the mean percentage methylation by experimental group, with standard error bars. This was obtained for each animal by averaging the percentage methylation across all CpG sites in each region (*Glt1* CpG island: seven CpGs; *Glt1* proximal promoter: three CpGs and *Glt1* distal shore: two CpGs). For each .of the three timepoints for tissue collection (6, 12 and 24 h post-hypoxia): naïve (*n* = 3), sham (*n* = 3) and hypoxia–ischaemia (*n* = 8).

### Hypoxia–ischaemia induces an early neuroinflammatory response in both cortex and hippocampus

**Figure 5.**
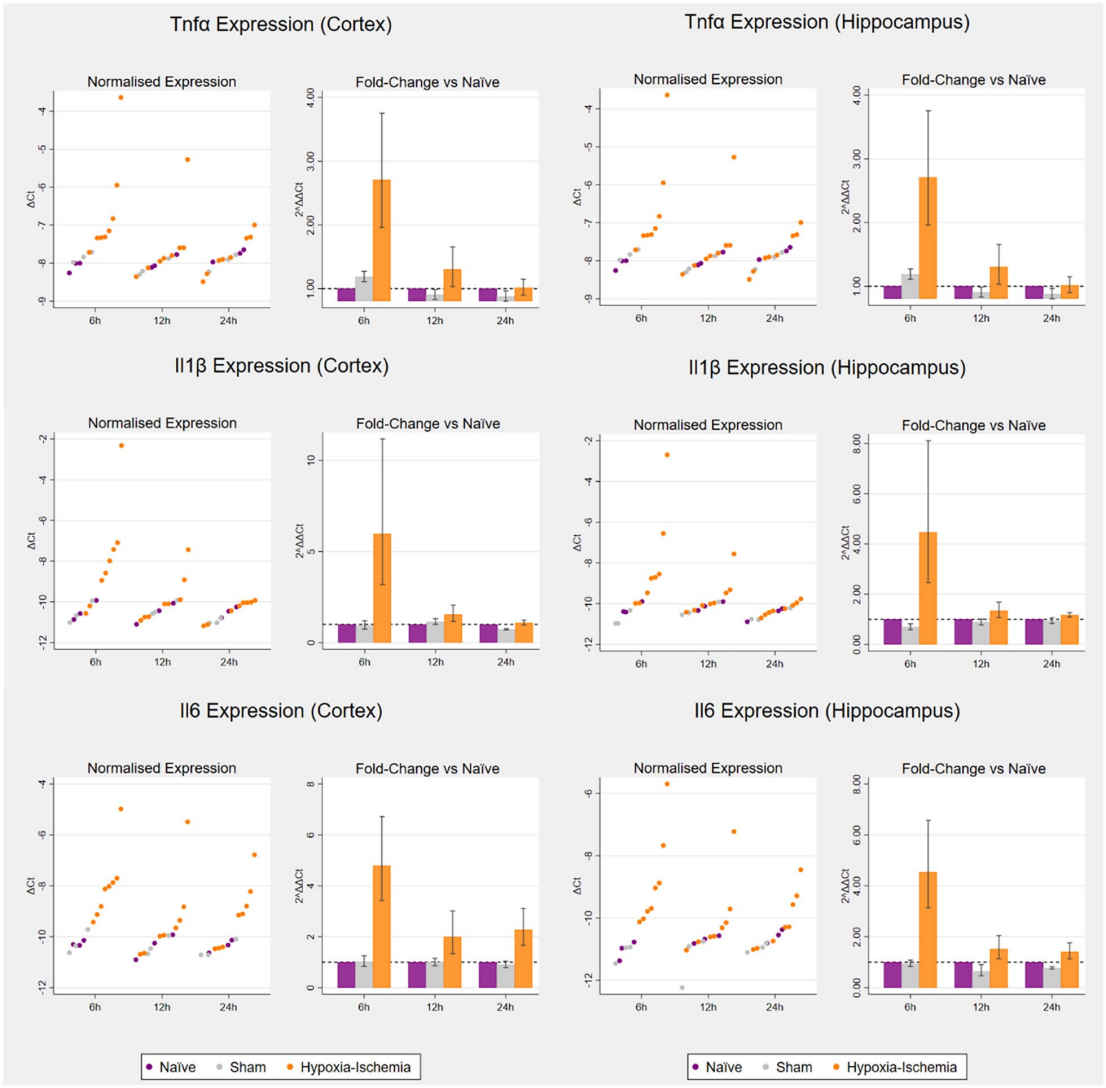
Cytokine expression following hypoxia–ischaemia in the cortex (left) and hippocampus (right)

There was evidence that transcription of all three pro-inflammatory cytokines (*Tnfα*, *Il1β* and *Il6*) was elevated 6 h after hypoxia–ischaemia in both cortex and hippocampus compared to naïve and sham groups, with a trend for a bimodal distribution (Table 3, Figure 5). Overall, group effects for all cytokines in both regions at 6 h surpassed Benjamini–Hochberg FDR (Supplementary Section 1.2.1.4 in the Supplementary Material).

**Table 3.** Cytokine expression: primary analyses.

Tissue	Gene	Timepoint (h)	*p*	Post hoc Dunn’s *p*
Cortex	*Tnfα*	6	0.025	N (*p* = 0.020) and S (*p* = 0.011) ≠ HI
		12	0.766	
		24	0.155	
	*Il1β*	6	0.034	N (*p* = 0.030) and S (*p* = 0.013) ≠ HI
		12	0.752	
		24	0.227	
	*Il6*	6	0.008	N (*p* = 0.008) and S (*p* = 0.006) ≠ HI
		12	0.433	
		24	0.129	
Hippocampus	*Tnfα*	6	0.010	N (*p* = 0.003) and S (*p* = 0.022) ≠ HI
		12	0.375	
		24	0.836	
	*Il1β*	6	0.014	N (*p* = 0.032) and S (*p* = 0.004) ≠ HI
		12	0.399	
		24	0.358	
	*Il6*	6	0.008	N (*p* = 0.008) and S (*p* = 0.006) ≠ HI
		12	0.110	
		24	0.135	

For each of the three timepoints for tissue collection (6, 12 and 24 h post-hypoxia): naïve (*n* = 3), sham (*n* = 3) and hypoxia–ischaemia (*n* = 8). N: naïve; S: sham; HI: hypoxia–ischaemia.

The scatterplots on the left reproduce untransformed Δ*Ct* values on the logarithmic scale. The bar charts on the right represent 2^ΔΔCt^ values, that is, the fold change on the linear scale in relation to respective age-matched controls, with potentiated means, potentiated asymmetric standard error bars and age-matched controls set as *y* = 1. For each of the three timepoints for tissue collection (6, 12 and 24 h post-hypoxia): naïve (*n* = 3), sham (*n* = 3) and hypoxia–ischaemia (*n* = 8).

## Discussion

In the newborn rat brain, moderate-severe hypoxia–ischaemia did not significantly alter transcription and promoter DNA methylation of the main glutamate transporter *Glt1* in bulk tissue from the cortex and hippocampus. On the other hand, transcription of three key pro-inflammatory cytokines (*Tnfα*, *Il1β* and *Il6*) increased at 6 h in both brain regions. This points to an early inflammatory response in the newborn brain and supports further work focusing on inflammation as a key pathway following hypoxia–ischaemia.

The early timepoints for sacrifice in this pilot study prevented direct assessment of brain tissue loss and of changes in levels of established markers of neuronal and glial injury. Following hypoxia–ischaemia, astrocytes nearby injured tissue become reactive due to the locally rising levels of danger associated molecular patterns, cytokines and reactive oxygen species (Derugin et al., 2000; Li et al., 2017). One of the hallmarks of astrogliosis is rapid accumulation of intermediate filament protein, glial fibrillary acidic protein (GFAP) (Eng, 1985; Magaki et al., 2018). The accumulation of GFAP protein is well documented in rodent models in the days and weeks after hypoxia–ischaemia (Bona et al., 1999; Burtrum and Silverstein, 1994; Towfighi et al., 1995; Xiong et al., 2009), as well as in nonhuman primates (McAdams et al., 2017). In addition, loss of *Mbp* mRNA and oligodendrocyte death have been shown in the subcortical white matter, corpus callosum and deep grey matter in the days after hypoxia–ischaemia (Follett et al., 2000; Liu et al., 2002; Reinboth et al., 2016; Skoff et al., 2001). The early transcriptional changes in *Gfap* and *Mbp* observed in this pilot study point to astroglial and oligodendroglial responses starting in the first hours after hypoxia–ischaemia. These early transcriptional changes are in the expected direction of effect and can be hypothesised to precede the substantial changes in protein levels known to occur during injury progression. As such, these changes point to some of the known neuropathological features of HIE, that is, astrogliosis and myelin injury, and therefore, support clinical relevance of the model.

MAP2 protein is an early and sensitive marker of the distribution, time course and severity of neuronal injury after hypoxia–ischaemia (Lingwood et al., 2008; Malinak and Silverstein, 1996; Ota et al., 1997 van den (Blomgren et al., 1995; Dawson and Hallenbeck, 1996; Fukamachi et al., 2001; Gilland et al., 1998; McRae et al., 1995; Puka-Sundvall et al., 2000; Van den Tweel et al., 2006). The lack of changes in *Map2* mRNA levels may be due to early timepoints in the study and/or partial uncoupling with protein levels due to post-translational regulation. This explanation is supported by the finding that MAP2 protein levels increase in the developing mouse from birth to P21, while mRNA levels remain more constant (Charrière-Bertrand et al., 1991).

In this pilot study, there was no significant evidence that hypoxia–ischaemia altered transcriptional regulation of the main glutamate transporter, *Glt1*, in either cortex or hippocampus. Alongside the small sample size, the use of bulk tissue may be particularly penalising in terms of power for *Glt1*, which is selectively expressed by astrocytes at this gestational age (Furuta et al., 1997). It is possible that changes may become detectable only after astrocyte isolation by magnetic-activated cell sorting (MACS) or fluorescence-activated cell sorting (FACS). Moreover, GLT1 increases three- to five-fold throughout the central nervous system from the second postnatal week onwards, and baseline levels may be low at P7 (Furuta et al., 1997; Levy et al., 1995; Rothstein et al., 1994; Shibata et al., 1996; Sutherland et al., 1996; Ullensvang et al., 1997). While the P7 Vannucci model benefits from decades of published studies, the more recent P10 model may be particularly relevant for studies of *Glt1* (Patel et al., 2015).

Assessing DNA methylation remained relevant even in the absence of obvious transcriptional changes, since epigenetic changes may have preceded latent transcriptional effects measurable after 24 h. We found no evidence that hypoxia–ischaemia altered DNA methylation at any of the candidate regions in *Glt1* promoter in the cortex. Crucially, the good quality *Peg3* data suggest that a lack of significant DNA methylation changes for *Glt1* reflects true negative findings with these experimental conditions and sample size rather than a technical failure to detect effects. CpG islands are nearly always unmethylated (Deaton and Bird, 2011; Jones, 2012; Rivera and Ren, 2013; Saxonov et al., 2006; Trerotola et al., 2015; Ziller et al., 2013), with few exceptions (e.g. imprinted genes) (Jones, 2012). Accordingly, the *Glt1* CpG island is unmethylated in the P1 rat cortex and cerebellum, with established inducers of *Glt1* leaving DNA methylation unaffected (Perisic et al., 2010, 2012). On the other hand, hypermethylation of the *EAAT2* CpG island with *EAAT2* silencing has been previously reported in human glioma cell lines (Zschocke et al., 2007). In this study, the CpG island of the *Glt1* promoter was found to be unmethylated in the P7 cortex, with no evidence of significant changes after hypoxia–ischaemia.

Shore regions located up to 2 kb from the transcription start site show lower CpG density and more dynamic methylation patterns across tissues and regions, with functions that remain poorly understood (Bae et al., 2016; Ferreira and Esteller, 2018; Illingworth and Bird, 2009; Irizarry et al., 2009; Jones, 2012; Schmidl et al., 2009). The *Glt1* shore region has been shown to have dynamic methylation patterns, responsible for differences in gene expression in different brain regions (cortex versus cerebellum), both at baseline and in response to *Glt1* inducers (Perisic et al., 2010, 2012). This higher epigenetic plasticity allows the region to act as an epigenetically regulated enhancer in the cortex, as demonstrated via a reporter gene assay in rat astrocytes (Perisic et al., 2010, 2012). In humans, the region homologous to the proximal promoter has been identified as differentially methylated in a saliva-based EWAS of very preterm versus healthy term newborns (Sparrow et al., 2016), potentially reflecting changes induced by the profound stress of prematurity. In this pilot study, the small changes identified in blood in the CpG island and proximal promoter regions were below the 5% detection limit of pyrosequencing (Hogg et al., 2014; Mikeska et al., 2011) and likely not biologically relevant. Further insights into potential epigenetic changes triggered in the early stages following acute hypoxia–ischaemia may be gained by measuring levels of methylation regulatory proteins in the acute phases. Moreover, epigenetic mechanisms act in concert with each other to alter chromatin structure and accessibility for transcription and translation (Bernstein et al., 2010; ENCODE Project Consortium, 2012; Kundaje et al., 2015; Lawrence et al., 2016; Zhang and Reinberg, 2001). Perhaps the effects of hypoxia–ischaemia are manifested in more transient histone modifications, reflecting the dynamic regulation of transcription rather than long-term gene silencing (Dulac, 2010).

As expected, hypoxia–ischaemia caused an upregulation of three key pro-inflammatory cytokines (*Tnfα*, *Il1β* and *Il6*) at 6 h following hypoxia–ischaemia. This finding aligns with previous evidence of early upregulation in the ipsilateral versus contralateral rat brain hemispheres (Bona et al., 1999; Hagberg et al., 1996; Szaflarski et al., 1995). The finding also aligns with evidence of increased *Tnfα* and *Il1β* mRNA levels 3 h post-hypoxia in the ipsilateral hemisphere compared to sham rats, with simultaneous infiltration of peripheral neutrophils and microglial activation (Bonestroo et al., 2013). Importantly, IL1β and IL6 protein levels and activity have been shown to accompany mRNA upregulation in relevant rodent models (Hagberg et al., 1996; Hu et al., 2005). Adding to previous evidence, the current study features three time points in the early phases of injury and introduces a regional dimension rather than analysing hemispheres in bulk, with detection of a transient inflammatory response in two of the brain regions most vulnerable to hypoxia–ischaemia. A main limitation was the inability to identify cellular sources and targets, which may be achieved in future work via MACS or FACS. Based on previous evidence, the main cellular sources of cytokines are likely to be activated microglia, infiltrating peripheral immune cells and reactive astrocytes (Hagberg et al., 2015; McAdams and Juul, 2012; Ziemka-Nalecz et al., 2017). The bimodal distribution observed for the cytokines and *Gfap* is in alignment with the bimodal distribution of injury observed in the largest study to date featuring this model and merging multiple smaller studies to achieve a sample size of over 600 rats (Wood et al., 2020). This may indicate existence of two distinct population of rats with differences in underlying initial characteristics (e.g. blood flow) potentially contributing to differences in brain injury (Vannucci et al., 1988; Wood et al., 2016b).

Accumulating preclinical evidence supports the notion that hypoxia–ischaemia triggers an early inflammatory response which contributes to injury of neurons, axons and oligodendrocytes depending on stage of brain development (Ziemka-Nalecz et al., 2017). In humans, evidence of a causal relationship between hypoxia–ischaemia and inflammation comes from observation that cerebral ischaemia induced by circulatory arrest during cardiac surgery in the newborn triggers a systemic inflammatory response within minutes (Algra et al., 2013). These findings support further research into immunomodulatory therapies for neuroprotection and into biomarkers for rapid stratification of HIE newborns by inflammatory status (Aden et al., 2010; Girard et al., 2012; Juul et al., 2018; Liu et al., 2015). Building on this and previous studies, future work could assess whether hypoxia–ischaemia alters cytokine methylation, and whether epigenetic inflammatory biomarkers from the periphery provide information on the ongoing brain injury and translate to clinical use for HIE newborns.

## Supplemental Material

sj-docx-1-bna-10.1177_23982128221097568 – Supplemental material for Regulation of glutamate transport and neuroinflammation in a term newborn rat model of hypoxic–ischaemic brain injury Click here for additional data file. Supplemental material, sj-docx-1-bna-10.1177_23982128221097568 for Regulation of glutamate transport and neuroinflammation in a term newborn rat model of hypoxic–ischaemic brain injury by Silvia Pregnolato, Hemmen Sabir, Karen Luyt, Kira DA Rienecker, Anthony R Isles and Elavazhagan Chakkarapani in Brain and Neuroscience Advances
