# Supplementary material for: Regulation of glutamate transport and neuroinflammation in a term newborn rat model of hypoxic–ischaemic brain injury
Source: Brain Neurosci Adv. 2022 May 20;6:23982128221097568. doi: 10.1177/23982128221097568 (PMC9125068; doi:10.1177/23982128221097568)
Supplement: sj-docx-1-bna-10.1177_23982128221097568 – Supplemental material for Regulation of glutamate transport and neuroinflammation in a term newborn rat model of hypoxic–ischaemic brain injury [file sj-docx-1-bna-10.1177_23982128221097568.docx]

# Supplemental Materials

## Supplemental Methods

### qPCR primers and protocol

Table S1. qPCR primers

| **Primer Name** | **Primer Sequence** | **Exon Boundary** | **Product Size (bp)** |
| --- | --- | --- | --- |
| *glt1_F* | CGTGGGCCTCATCATTCATG | 1-2 | 151 |
| *glt1_R* | GGTGACAGGCAAAGTTCCAG |  |  |
| *tnfα_F* | CCACCACGCTCTTCTGTCTA | 1-2 | 90 |
| *tnfα_R* | TGGAACTGATGAGAGGGAGC |  |  |
| *il1β _F* | GGGATGATGACGACCTGCTA | 5-6 | 192 |
| *il1β_R* | TGTCGTTGCTTGTCTCTCCT |  |  |
| *il6_F* | CTGCTCTGGTCTTCTGGAGT | 4-5 | 180 |
| *il6_R* | AGAGCATTGGAAGTTGGGGT |  |  |
| *mbp_F* | CGCAGAGGACCCAAGATGAA | 3-4 | 195 |
| *mbp_R* | CAGGATTCGGGAAGGCTGA |  |  |
| *gfap_F* | TAAGCTAGCCCTGGACATCG | 2-3 | 105 |
| *gfap_R* | CGGATCTGGAGGTTGGAGAA |  |  |
| *map2_F* | TGAACAAGAGAAGGAGGCCC | 3-4 | 174 |
| *map2_R* | TGTTTAAACGCACTGGGAGC |  |  |
| *hprt_F* | AGGACCTCTCGAAGTGTTGG | 7-9 | 137 |
| *hprt_R* | CCACTTTCGCTGATGACACA |  |  |
| *b2m_F* | TCTGGTGCTTGTCTCTCTGG | 1-2 | 126 |
| *b2m_R* | TGGAACTGAGACACGTAGCA |  |  |

Table S2. qPCR recipe

| **Reagent** | **Volume (1 reaction)** |
| --- | --- |
| SensiFAST SYBR Green | 12.5 µl |
| Forward Primer | 1.75 µl |
| Reverse Primer | 1.75 µl |
| Nuclease-Free Water | 4 µl |
| cDNA Template/No Template Control | 5 µl |
| Total | 25 µl |

Table S3. qPCR protocol

| **PCR Cycle Step** |  | **Temperature** | **Time** | **No. Cycles** |
| --- | --- | --- | --- | --- |
| Taq Heat Activation |  | 95°C | 10 min |  |
| Denaturation |  | 95°C | 20 sec | x 40 |
| Annealing |  | 60°C | 20 sec |  |
| Extension |  | 72°C | 20 sec |  |

### Two-step PCR and pyrosequencing primers and protocol

Table S4. DNA methylation primers

These include forward (F) and reverse (R) PCR primers, and pyrosequencing (S) primer. Whenever it was not possible to avoid a CpG site, degenerate primers containing all four bases (N, in red) at the site were used. The common primer sequence is shown in blue. The quality score was automatically assigned by the Pyromark Assay Design 2.0 software

| **Primer** | **Sequence** | **PCR#2 Product Size** | **Score** |
| --- | --- | --- | --- |
| *common_biotin* | Btn - CGCCAGGGTTTTCCCAGTCACGAC | 24 |  |
| *glt1_dist_F* | GGTTGAAGAGAATATAAAGTTGAT | 399 + 24 = 423 | 68 |
| *glt1_dist_R* | CGCCAGGGTTTTCCCAGTCACGACACATCTTCNCTTCTTTACATCCACTACA |  |  |
| *glt1_dist_S* | AAATAGGAGAGGGTG |  |  |
| *glt1_prox_F* | GTTATTATTATGTAAAGTTGGGTATGAGAT | 154 + 24 = 178 | 66 |
| *glt1_prox_R* | CGCCAGGGTTTTCCCAGTCACGACCCACTCCCCAAACCAAATACTAAAA |  |  |
| *glt1_prox_S* | AGTTTATTTGTTTTTGTATATTTT |  |  |
| *glt1_isl_F* | GGTGATGTTAGTTTTGGATAAAAATAGAGA | 272 + 24 = 296 | 63 |
| *glt1_isl_R* | CGCCAGGGTTTTCCCAGTCACGACCCTAACCNCCACCTATATCTATACTTC |  |  |
| *glt1_isl_S* | GGGGTTAAATTTTGTAATTTTT |  |  |
| *peg3_F* | TTGTTGTAGANGTTGGGGAGTTAAGA | 125+24=149 | 82 |
| *peg3_R* | CGCCAGGGTTTTCCCAGTCACGACACAATCTAATACACCCACACTAA |  |  |
| *peg3_S* | TGGGGAGTTAAGAGT |  |  |

Table S5. Two-step PCR recipe

All PCR assays were individually optimised prior to the experiment, and an annealing temperature of 57°C was found to be appropriate for all assays. The steps for the two PCRs are identical except for:

1. The template was bcDNA in PCR#1, and a 1:6 dilution of PCR#1 products in PCR#2
2. The gene/region-specific reverse primer was replaced with the biotinylated common primer in PCR#2
3. Volumes were doubled in PCR#2
4. Three technical replicates per sample were run in PCR#2

| **PCR Reagents** | **Volume for one reaction** | **Working Concentration** |
| --- | --- | --- |
| Nuclease-Free Water | 6.175 µl |  |
| Buffer | 1 µl | 10X |
| dNTPs | 0.8 µl | 2 mM |
| F primer | 0.3 µl | 10 µM |
| R primer | 0.3 µl | 10 µM |
| HotStart Taq Polymerase | 0.125 µl | 5 U/µl |

Table S6. Two-step PCR protocol

| **PCR Cycle Step** | **Temperature** | **Time** | **No. Cycles** |
| --- | --- | --- | --- |
| Taq Heat Activation | 95°C | 15 min |  |
| Denaturation | 95°C | 30 sec | X 25 |
| Annealing | 57°C | 60 sec |  |
| Extension | 72°C | 40 sec |  |
| Final Extension | 72°C | 10 min |  |
| Hold | 4°C | Indefinitely |  |

## Supplemental Results

### Gene Expression Analysis

#### Housekeeping Genes

There was no significant evidence that transcription of either *B2m* (6h: p=0.252; 12h: 0.859; 24h: p=0.297) or *Hprt* (6h: p=0.492; 12h: p=0.062; 24h: p=0.628)was affected by group.

#### Markers of Injury

##### Cortex

Table S7. Markers of injury (cortex): primary analyses

For each of the three timepoints for tissue collection (6h, 12h, 24h post-hypoxia): naïve (n=3), sham (n=3), hypoxia-ischemia (n=8). N: naïve; S: sham; HI: hypoxia-ischaemia.

| **Gene** | **Variable** | **p** | ***Post hoc* Dunn’s p** |
| --- | --- | --- | --- |
| ***Mbp*** | *6h* | **0.031** | N (p=0.086) and S (p=0.006) ≠ HI |
|  | *12h* | **0.041** | S (p=0.006) ≠ HI |
|  | *24h* | 0.064 | N (p=0.021) and S (p=0.047) ≠ HI |
| ***Gfap*** | *6h* | **0.021** | N (p=0.006) and S (p=0.035) ≠ HI |
|  | *12h* | **0.016** | N (p=0.005) and S (p=0.024) ≠ HI |
|  | *24h* | 0.151 |  |
| ***Map2*** | *6h* | 0.995 |  |
|  | *12h* | 0.254 |  |
|  | *24h* | 0.257 |  |

Figure S1. *Mbp* expression (cortex)

The scatterplots on the left reproduce untransformed ΔCt values on the logarithmic scale, with means and standard error bars. The bar charts on the right represent 2^ΔΔCt^ fold change on the linear scale in relation to respective age-matched controls, with potentiated means, potentiated asymmetric standard error bars and age-matched controls set as y=1. For each of the three timepoints for tissue collection (6h, 12h, 24h post-hypoxia): naïve (n=3), sham (n=3), hypoxia-ischemia (n=8).


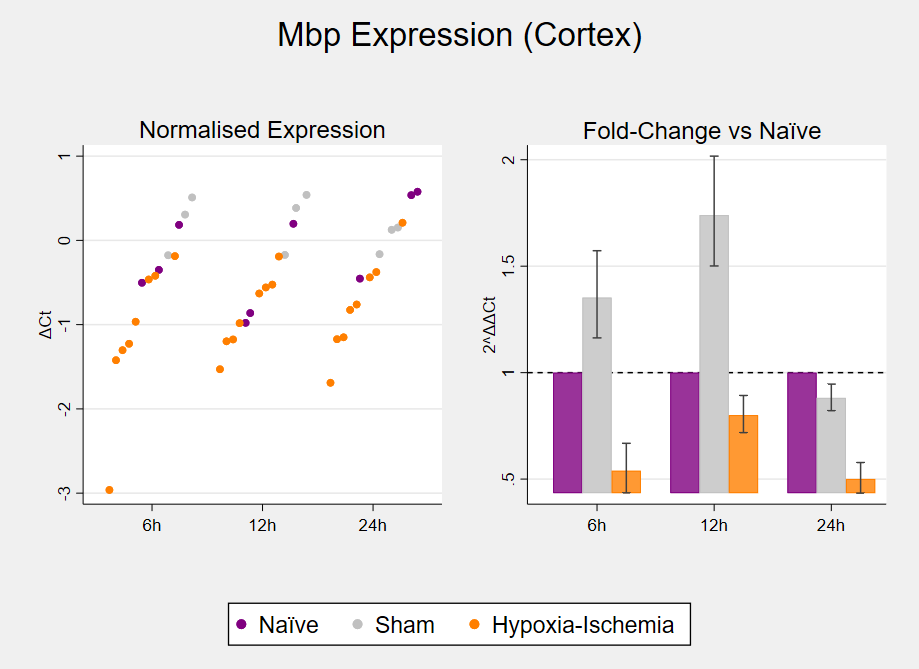


Figure S2. *Gfap* expression (cortex)

The scatterplots on the left reproduce untransformed ΔCt values on the logarithmic scale, with means and standard error bars. The bar charts on the right represent 2^ΔΔCt^ fold change on the linear scale in relation to respective age-matched controls, with potentiated means, potentiated asymmetric standard error bars and age-matched controls set as y=1. For each of the three timepoints for tissue collection (6h, 12h, 24h post-hypoxia): naïve (n=3), sham (n=3), hypoxia-ischemia (n=8).

**
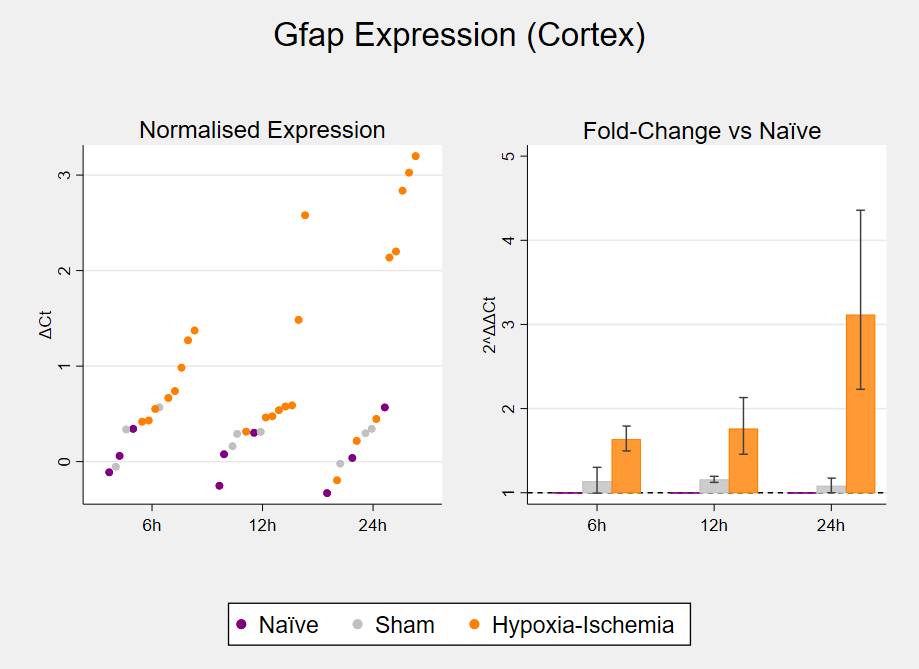
**

Figure S3. *Map2* expression (cortex)

The scatterplots on the left reproduce untransformed ΔCt values on the logarithmic scale, with means and standard error bars. The bar charts on the right represent 2^ΔΔCt^ fold change on the linear scale in relation to respective age-matched controls, with potentiated means, potentiated asymmetric standard error bars and age-matched controls set as y=1. For each of the three timepoints for tissue collection (6h, 12h, 24h post-hypoxia): naïve (n=3), sham (n=3), hypoxia-ischemia (n=8).

**
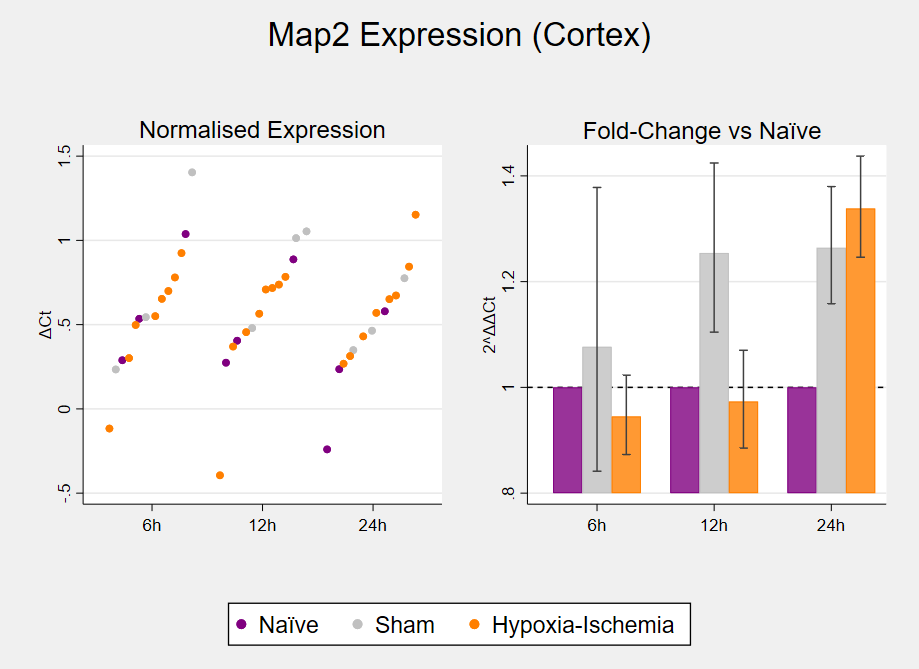
**

Table S8. Markers of injury (cortex): secondary analyses

For each of the three timepoints for tissue collection (6h, 12h, 24h post-hypoxia): naïve (n=3), sham (n=3), hypoxia-ischemia (n=8). N: naïve; S: sham; HI: hypoxia-ischaemia.

| **Gene** | **Design of secondary analysis** | **Variable** | **p** |
| --- | --- | --- | --- |
| ***Mbp*** | *N vs S* | *6h* | 0.127 |
|  |  | *12h* | 0.127 |
|  |  | *24h* | 0.513 |
|  | *N/S vs HI* | *6h* | **0.014** |
|  |  | *12h* | **0.039** |
|  |  | *24h* | **0.020** |
| ***Gfap*** | *N vs S* | *6h* | 0.513 |
|  |  | *12h* | 0.275 |
|  |  | *24h* | 0.827 |
|  | *N/S vs HI* | *6h* | **0.007** |
|  |  | *12h* | **0.005** |
|  |  | *24h* | 0.053 |
| ***Map2*** | *N vs S* | *6h* | 0.827 |
|  |  | *12h* | 0.127 |
|  |  | *24h* | 0.275 |
|  | *N/S vs HI* | *6h* | >0.999 |
|  |  | *12h* | 0.439 |
|  |  | *24h* | 0.245 |

##### Hippocampus

Table S9. Markers of injury (hippocampus): primary analyses

For each of the three timepoints for tissue collection (6h, 12h, 24h post-hypoxia): naïve (n=3), sham (n=3), hypoxia-ischemia (n=8). N: naïve; S: sham; HI: hypoxia-ischaemia.

| **Gene** | **Variable** | **p** | ***Post hoc* Dunn’s p** |
| --- | --- | --- | --- |
| ***Mbp*** | *6h* | 0.860 |  |
|  | *12h* | 0.135 |  |
|  | *24h* | 0.110 |  |
| ***Gfap*** | *6h* | **0.018** | N (p=0.014) and S (p=0.010) ≠ HI |
|  | *12h* | 0.095 |  |
|  | *24h* | 0.151 |  |
| ***Map2*** | *6h* | 0.576 |  |
|  | *12h* | 0.658 |  |
|  | *24h* | 0.399 |  |

Figure S4. *Mbp* expression (hippocampus)

The scatterplots on the left reproduce untransformed ΔCt values on the logarithmic scale, with means and standard error bars. The bar charts on the right represent 2^ΔΔCt^ fold change on the linear scale in relation to respective age-matched controls, with potentiated means, potentiated asymmetric standard error bars and age-matched controls set as y=1. For each of the three timepoints for tissue collection (6h, 12h, 24h post-hypoxia): naïve (n=3), sham (n=3), hypoxia-ischemia (n=8).


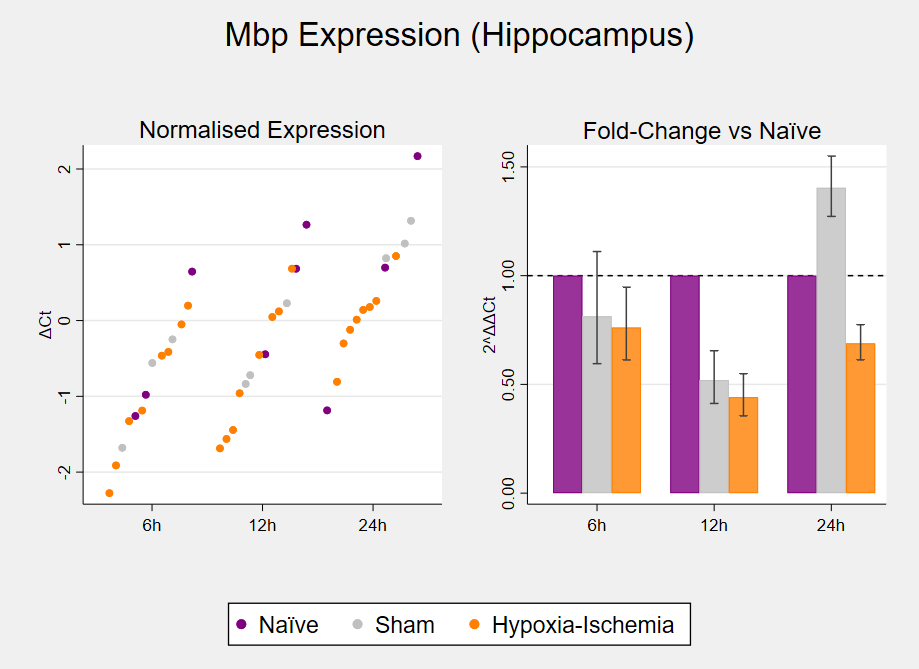


Figure S5. *Gfap* expression (hippocampus)

The scatterplots on the left reproduce untransformed ΔCt values on the logarithmic scale, with means and standard error bars. The bar charts on the right represent 2^ΔΔCt^ fold change on the linear scale in relation to respective age-matched controls, with potentiated means, potentiated asymmetric standard error bars and age-matched controls set as y=1. For each of the three timepoints for tissue collection (6h, 12h, 24h post-hypoxia): naïve (n=3), sham (n=3), hypoxia-ischemia (n=8).


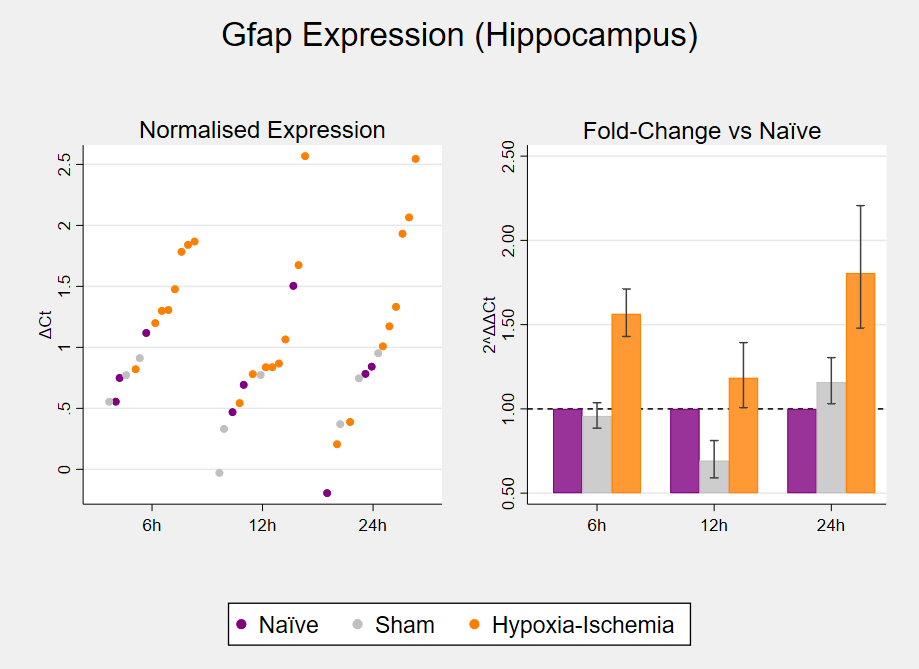


Figure S6. *Map2* expression (hippocampus)

The scatterplots on the left reproduce untransformed ΔCt values on the logarithmic scale, with means and standard error bars. The bar charts on the right represent 2^ΔΔCt^ fold change on the linear scale in relation to respective age-matched controls, with potentiated means, potentiated asymmetric standard error bars and age-matched controls set as y=1. For each of the three timepoints for tissue collection (6h, 12h, 24h post-hypoxia): naïve (n=3), sham (n=3), hypoxia-ischemia (n=8).


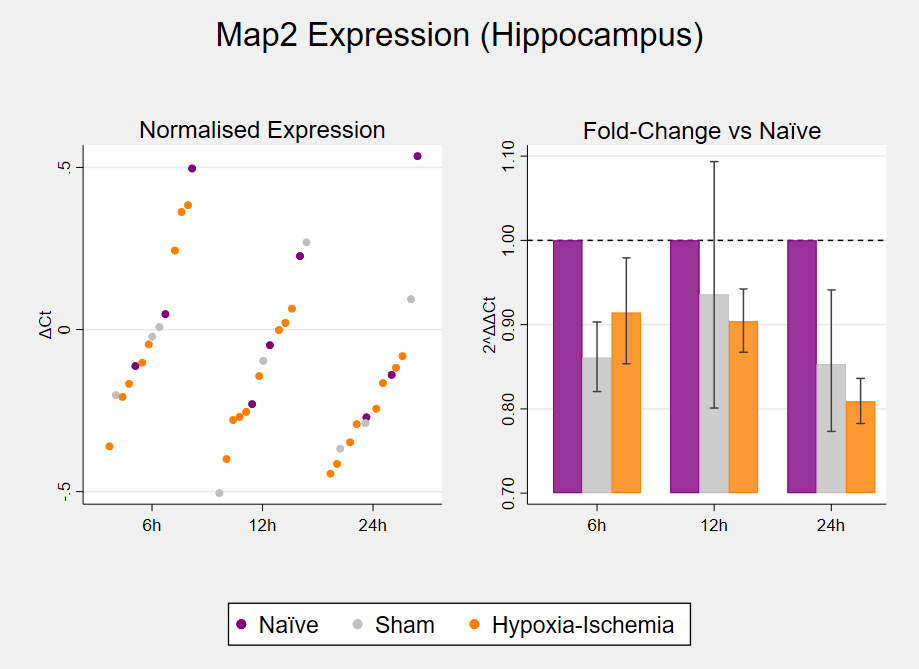


Table S10. Markers of injury (hippocampus): secondary analyses

For each of the three timepoints for tissue collection (6h, 12h, 24h post-hypoxia): naïve (n=3), sham (n=3), hypoxia-ischemia (n=8). N: naïve; S: sham; HI: hypoxia-ischaemia.

| **Gene** | **Design of secondary analysis** | **Variable** | **p** |
| --- | --- | --- | --- |
| ***Mbp*** | *N vs S* | *6h* | 0.827 |
|  |  | *12h* | 0.127 |
|  |  | *24h* | 0.513 |
|  | *N/S vs HI* | *6h* | 0.699 |
|  |  | *12h* | 0.121 |
|  |  | *24h* | 0.071 |
| ***Gfap*** | *N vs S* | *6h* | 0.827 |
|  |  | *12h* | 0.275 |
|  |  | *24h* | 0.827 |
|  | *N/S vs HI* | *6h* | **0.005** |
|  |  | *12h* | 0.053 |
|  |  | *24h* | 0.053 |
| ***Map2*** | *N vs S* | *6h* | 0.275 |
|  |  | *12h* | 0.827 |
|  |  | *24h* | 0.275 |
|  | *N/S vs HI* | *6h* | 0.699 |
|  |  | *12h* | 0.439 |
|  |  | *24h* | 0.302 |

#### Neuroinflammation

##### Cortex

Table S11. Inflammation (cortex): secondary analyses

For each of the three timepoints for tissue collection (6h, 12h, 24h post-hypoxia): naïve (n=3), sham (n=3), hypoxia-ischemia (n=8). N: naïve; S: sham; HI: hypoxia-ischaemia.

| **Gene** | **Design of secondary analysis** | **Variable** | **p** |
| --- | --- | --- | --- |
| ***Tnfα*** | *N vs S* | *6h* | 0.513 |
|  |  | *12h* | 0.513 |
|  |  | *24h* | 0.275 |
|  | *N/S vs HI* | *6h* | **0.007** |
|  |  | *12h* | 0.796 |
|  |  | *24h* | 0.071 |
| ***Il1β*** | *N vs S* | *6h* | 0.513 |
|  |  | *12h* | 0.827 |
|  |  | *24h* | 0.495 |
|  | *N/S vs HI* | *6h* | **0.010** |
|  |  | *12h* | 0.519 |
|  |  | *24h* | 0.156 |
| ***Il6*** | *N vs S* | *6h* | 0.827 |
|  |  | *12h* | 0.827 |
|  |  | *24h* | 0.513 |
|  | *N/S vs HI* | *6h* | **0.002** |
|  |  | *12h* | 0.197 |
|  |  | *24h* | 0.053 |

##### Hippocampus

Table S12. Inflammation (hippocampus): secondary analyses

For each of the three timepoints for tissue collection (6h, 12h, 24h post-hypoxia): naïve (n=3), sham (n=3), hypoxia-ischemia (n=8). N: naïve; S: sham; HI: hypoxia-ischaemia.

| **Gene** | **Design of secondary analysis** | **Variable** | **p** |
| --- | --- | --- | --- |
| ***Tnfα*** | *N vs S* | *6h* | 0.275 |
|  |  | *12h* | 0.275 |
|  |  | *24h* | 0.275 |
|  | *N/S vs HI* | *6h* | **0.003** |
|  |  | *12h* | 0.245 |
|  |  | *24h* | 0.897 |
| ***Il1β*** | *N vs S* | *6h* | 0.275 |
|  |  | *12h* | 0.275 |
|  |  | *24h* | 0.827 |
|  | *N/S vs HI* | *6h* | **0.005** |
|  |  | *12h* | 0.302 |
|  |  | *24h* | 0.156 |
| ***Il6*** | *N vs S* | *6h* | 0.827 |
|  |  | *12h* | 0.127 |
|  |  | *24h* | 0.051 |
|  | *N/S vs HI* | *6h* | **0.002** |
|  |  | *12h* | 0.071 |
|  |  | *24h* | 0.121 |

#### False Discovery Rate

Table S13. Gene expression in the cortex at 6h: findings surviving Benjamini-Hochberg False Discovery Rate (10%) correction (shaded)

| **Gene** | **Region** | **Timepoint** | **p-value for Group Effect (Primary Analyses)** | **Rank** | **Benjamini-Hochberg critical value** |
| --- | --- | --- | --- | --- | --- |
| *Il6* | Cortex | 6h | 0.008 | 1 | 0.014 |
| *Gfap* | Cortex | 6h | 0.021 | 2 | 0.029 |
| *Tnfα* | Cortex | 6h | 0.025 | 3 | 0.043 |
| *Mbp* | Cortex | 6h | 0.031 | 4 | 0.057 |
| *Il1β* | Cortex | 6h | 0.034 | 5 | 0.071 |
| *Glt1* | Cortex | 6h | 0.386 | 6 | 0.086 |
| *Map2* | Cortex | 6h | 0.995 | 7 | 0.100 |

Table S14. Gene expression in the cortex at 12h: no findings survived Benjamini-Hochberg False Discovery Rate (10%) correction

| **Gene** | **Region** | **Timepoint** | **p-value for Group Effect (Primary Analyses)** | **Rank** | **Benjamini-Hochberg critical value** |
| --- | --- | --- | --- | --- | --- |
| *Gfap* | Cortex | 12h | 0.016 | 1 | 0.014 |
| *Mbp* | Cortex | 12h | 0.041 | 2 | 0.029 |
| *Map2* | Cortex | 12h | 0.254 | 3 | 0.043 |
| *Il6* | Cortex | 12h | 0.433 | 4 | 0.057 |
| *Glt1* | Cortex | 12h | 0.562 | 5 | 0.071 |
| *Il1β* | Cortex | 12h | 0.752 | 6 | 0.086 |
| *Tnfα* | Cortex | 12h | 0.766 | 7 | 0.100 |

Table S15. Gene expression in the cortex at 24h: no findings survived Benjamini-Hochberg False Discovery Rate (10%) correction

| **Gene** | **Region** | **Timepoint** | **p-value for Group Effect (Primary Analyses)** | **Rank** | **Benjamini-Hochberg critical value** |
| --- | --- | --- | --- | --- | --- |
| *Mbp* | Cortex | 24h | 0.064 | 1 | 0.014 |
| *Il6* | Cortex | 24h | 0.129 | 2 | 0.029 |
| *Gfap* | Cortex | 24h | 0.151 | 3 | 0.043 |
| *Tnfα* | Cortex | 24h | 0.155 | 4 | 0.057 |
| *Glt1* | Cortex | 24h | 0.18 | 5 | 0.071 |
| *Il1β* | Cortex | 24h | 0.227 | 6 | 0.086 |
| *Map2* | Cortex | 24h | 0.257 | 7 | 0.100 |

Table S16. Gene expression in the hippocampus at 6h: findings surviving Benjamini-Hochberg False Discovery Rate (10%) correction (shaded)

| **Gene** | **Region** | **Timepoint** | **p-value for Group Effect (Primary Analyses)** | **Rank** | **Benjamini-Hochberg critical value** |
| --- | --- | --- | --- | --- | --- |
| *Il6* | Hippocampus | 6h | 0.008 | 1 | 0.014 |
| *Tnfα* | Hippocampus | 6h | 0.01 | 2 | 0.029 |
| *Il1β* | Hippocampus | 6h | 0.014 | 3 | 0.043 |
| *Gfap* | Hippocampus | 6h | 0.018 | 4 | 0.057 |
| *Glt1* | Hippocampus | 6h | 0.433 | 5 | 0.071 |
| *Map2* | Hippocampus | 6h | 0.576 | 6 | 0.086 |
| *Mbp* | Hippocampus | 6h | 0.86 | 7 | 0.100 |

Table S17. Gene expression in the hippocampus at 12h: no findings survived Benjamini-Hochberg False Discovery Rate (10%) correction

| **Gene** | **Region** | **Timepoint** | **p-value for Group Effect (Primary Analyses)** | **Rank** | **Benjamini-Hochberg critical value** |
| --- | --- | --- | --- | --- | --- |
| *Gfap* | Hippocampus | 12h | 0.095 | 1 | 0.014 |
| *Il6* | Hippocampus | 12h | 0.11 | 2 | 0.029 |
| *Mbp* | Hippocampus | 12h | 0.135 | 3 | 0.043 |
| *Tnfα* | Hippocampus | 12h | 0.375 | 4 | 0.057 |
| *Il1β* | Hippocampus | 12h | 0.399 | 5 | 0.071 |
| *Map2* | Hippocampus | 12h | 0.658 | 6 | 0.086 |
| *Glt1* | Hippocampus | 12h | 0.995 | 7 | 0.100 |

Table S18. Gene expression in the hippocampus at 24h: no findings survived Benjamini-Hochberg False Discovery Rate (10%) correction

| **Gene** | **Region** | **Timepoint** | **p-value for Group Effect (Primary Analyses)** | **Rank** | **Benjamini-Hochberg critical value** |
| --- | --- | --- | --- | --- | --- |
| *Mbp* | Hippocampus | 24h | 0.11 | 1 | 0.014 |
| *Il6* | Hippocampus | 24h | 0.135 | 2 | 0.029 |
| *Gfap* | Hippocampus | 24h | 0.151 | 3 | 0.043 |
| *Glt1* | Hippocampus | 24h | 0.262 | 4 | 0.057 |
| *Il1β* | Hippocampus | 24h | 0.358 | 5 | 0.071 |
| *Map2* | Hippocampus | 24h | 0.399 | 6 | 0.086 |
| *Tnfα* | Hippocampus | 24h | 0.836 | 7 | 0.100 |

### DNA methylation

#### Peg3

Table S19. *Peg3* – Percentage DNA methylation at individual CpG sites

| ***Peg3*: CpG Site** | **n** | **Cortex** | | **Blood** | |
| --- | --- | --- | --- | --- | --- |
|  |  | **Mean % DNAm** | **SD** | **Mean % DNAm** | **SD** |
| *1* | 42 | 48.13 | 1.52 | 46.78 | 1.90 |
| *2* | 42 | 46.95 | 1.50 | 45.10 | 1.72 |
| *3* | 42 | 49.43 | 1.57 | 48.03 | 2.29 |
| *4* | 42 | 50.75 | 2.03 | 50.95 | 2.91 |
| *5* | 42 | 51.10 | 1.37 | 50.83 | 1.85 |

Figure S7. *Peg3* – Percentage DNA methylation at individual CpG sites

The scatterplots reproduce percentage methylation at each CpG site, with means and standard error bars. For each of the three timepoints for tissue collection (6h, 12h, 24h post-hypoxia): naïve (n=3), sham (n=3), hypoxia-ischemia (n=8).


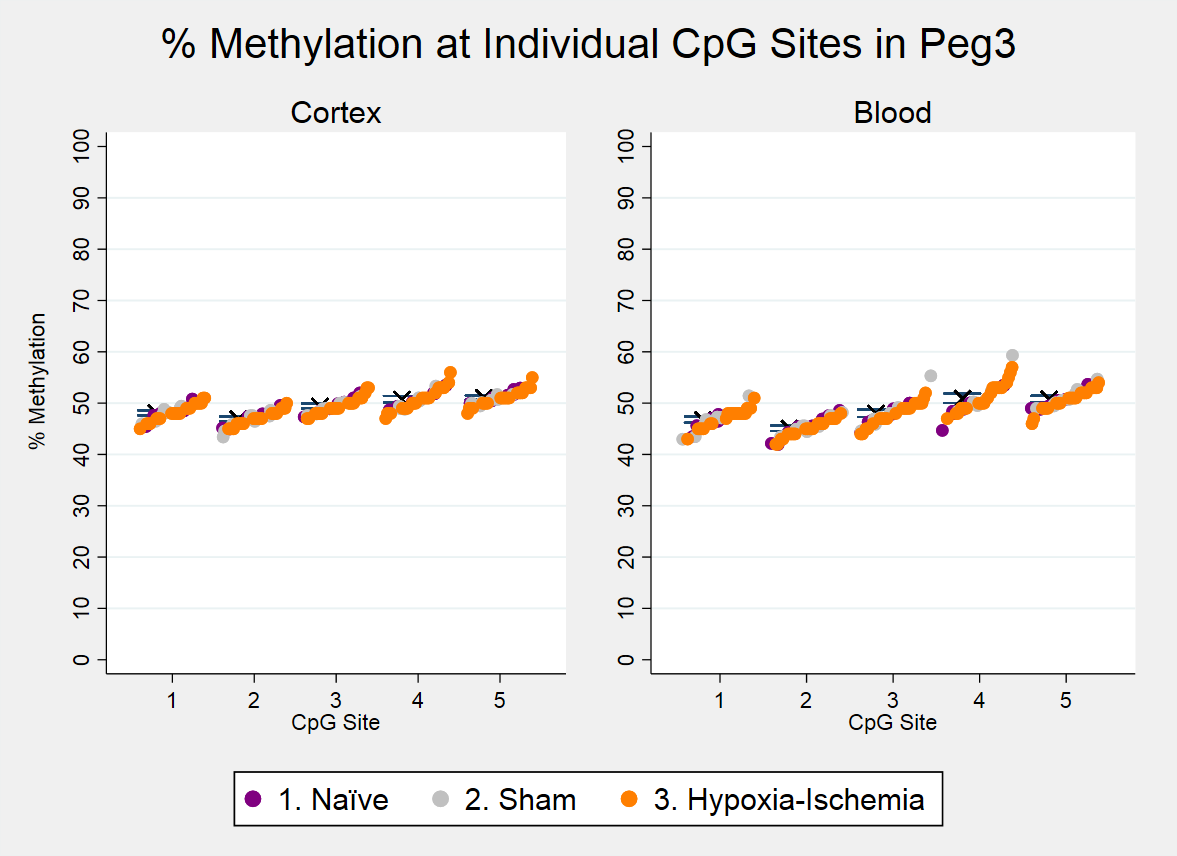


#### Glutamate Transporter

##### CpG island

Table S20. *Glt1* (CpG island) – Percentage DNA methylation at individual CpG sites

| ***Glt1* (CpG island): CpG Site** | **n** | **Cortex** | | **Blood** | |
| --- | --- | --- | --- | --- | --- |
|  |  | **Mean % DNAm** | **SD** | **Mean % DNAm** | **SD** |
| *1* | 42 | 0.22 | 0.42 | 4.15 | 3.67 |
| *2* | 42 | 1.78 | 1.38 | 3.79 | 2.67 |
| *3* | 42 | 0.25 | 0.50 | 0.94 | 1.71 |
| *4* | 42 | 0.08 | 0.28 | 0.67 | 1.24 |
| *5* | 42 | 0.72 | 2.17 | 2.06 | 2.32 |
| *6* | 42 | 2.75 | 1.38 | 5.33 | 3.41 |
| *7 (EAAT2 -181)* | 42 | 0.36 | 0.68 | 2.58 | 2.97 |

Figure S8. *Glt1* (CpG island) – Percentage DNA methylation at individual CpG sites

The scatterplots reproduce percentage methylation at each CpG site, with means and standard error bars. For each of the three timepoints for tissue collection (6h, 12h, 24h post-hypoxia): naïve (n=3), sham (n=3), hypoxia-ischemia (n=8).


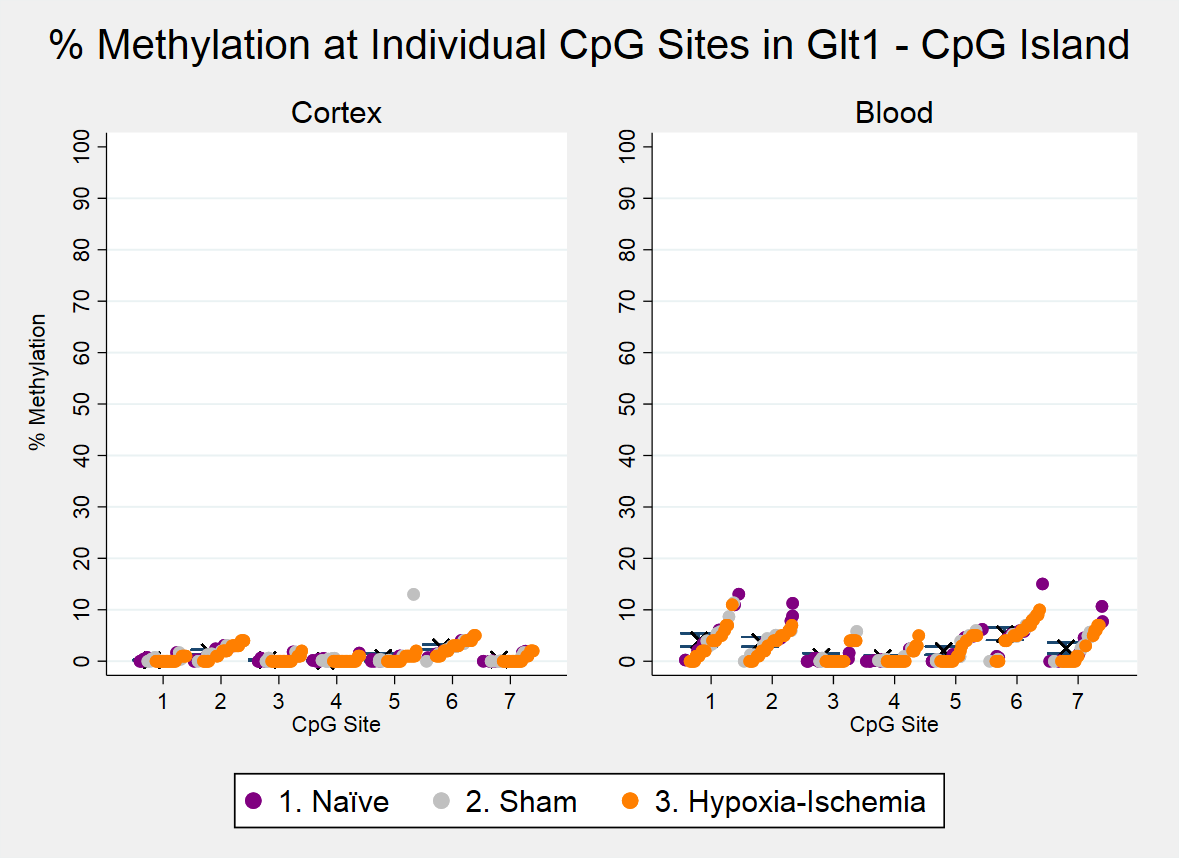


Table S21. *Glt1* (CpG island): secondary analyses

For each of the three timepoints for tissue collection (6h, 12h, 24h post-hypoxia): naïve (n=3), sham (n=3), hypoxia-ischemia (n=8). N: naïve; S: sham; HI: hypoxia-ischaemia.

| **Gene** | **Tissue** | **Design of secondary analysis** | **Variable** | **p** |
| --- | --- | --- | --- | --- |
| ***Glt1* (CpG island)** | ***Cortex*** | *N vs S* | *6h* | >0.999 |
|  |  |  | *12h* | 0.487 |
|  |  |  | *24h* | 0.817 |
|  |  | *N/S vs HI* | *6h* | 0.797 |
|  |  |  | *12h* | 0.626 |
|  |  |  | *24h* | 0.368 |

##### Proximal promoter

Of note, CpG 3 coincides with a CpG site (cg21163960) that is differentially methylated in the saliva of very preterm newborns vs healthy term newborns in an EWAS (Sparrow et al., 2016).

Table S22. *Glt1* (proximal promoter) – Percentage DNA methylation at individual CpG sites

| ***Glt1* (proximal promoter): CpG Site** | **n** | **Cortex** | | **Blood** | |
| --- | --- | --- | --- | --- | --- |
|  |  | **Mean % DNAm** | **SD** | **Mean % DNAm** | **SD** |
| *1* | 42 | 5.52 | 1.09 | 13.74 | 2.57 |
| *2* | 42 | 4.95 | 1.27 | 11.52 | 2.92 |
| *3* | 42 | 2.29 | 1.53 | 17.90 | 3.11 |

Figure S9. *Glt1* (proximal promoter) – Percentage DNA methylation at individual CpG sites

The scatterplots reproduce percentage methylation at each CpG site, with means and standard error bars. For each of the three timepoints for tissue collection (6h, 12h, 24h post-hypoxia): naïve (n=3), sham (n=3), hypoxia-ischemia (n=8).

**
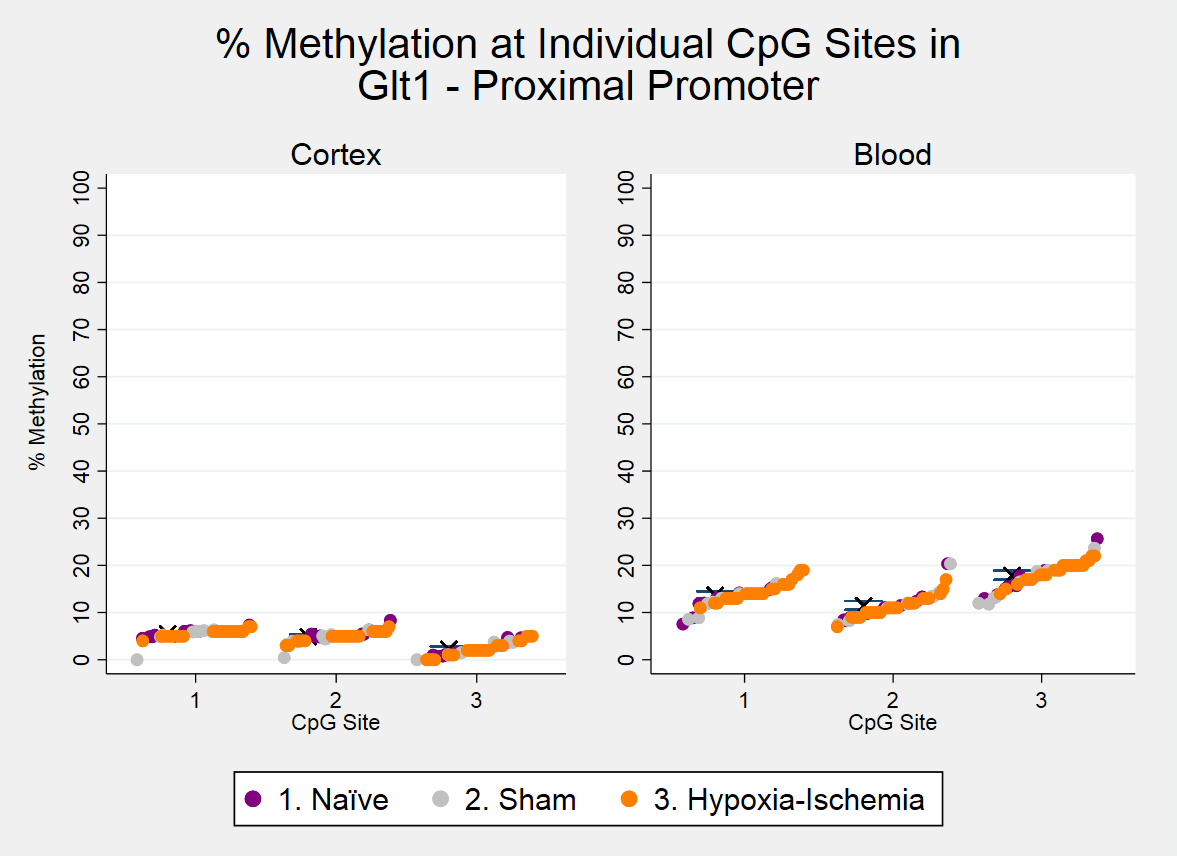
**

Table S23. *Glt1* (proximal promoter): secondary analyses

For each of the three timepoints for tissue collection (6h, 12h, 24h post-hypoxia): naïve (n=3), sham (n=3), hypoxia-ischemia (n=8). N: naïve; S: sham; HI: hypoxia-ischaemia.

| **Gene** | **Tissue** | **Design of secondary analysis** | **Variable** | **p** |
| --- | --- | --- | --- | --- |
| ***Glt1* (proximal promoter)** | ***Cortex*** | *N vs S* | *6h* | 0.121 |
|  |  |  | *12h* | 0.822 |
|  |  |  | *24h* | >0.999 |
|  |  | *N/S vs HI* | *6h* | 0.845 |
|  |  |  | *12h* | >0.999 |
|  |  |  | *24h* | 0.694 |

##### Distal shore

Table S24. *Glt1* (distal shore) – Percentage DNA methylation at individual CpG sites

| ***Glt1* (distal promoter): CpG Site** | **n** | **Cortex** | | **Blood** | |
| --- | --- | --- | --- | --- | --- |
|  |  | **Mean % DNAm** | **SD** | **Mean % DNAm** | **SD** |
| *1* | 42 | 15.26 | 3.44 | 57.40 | 5.23 |
| *2* | 42 | 15.43 | 3.82 | 42.14 | 4.21 |

**Figure S10. *Glt1* (distal shore) – Percentage DNA methylation at individual CpG sites**

**
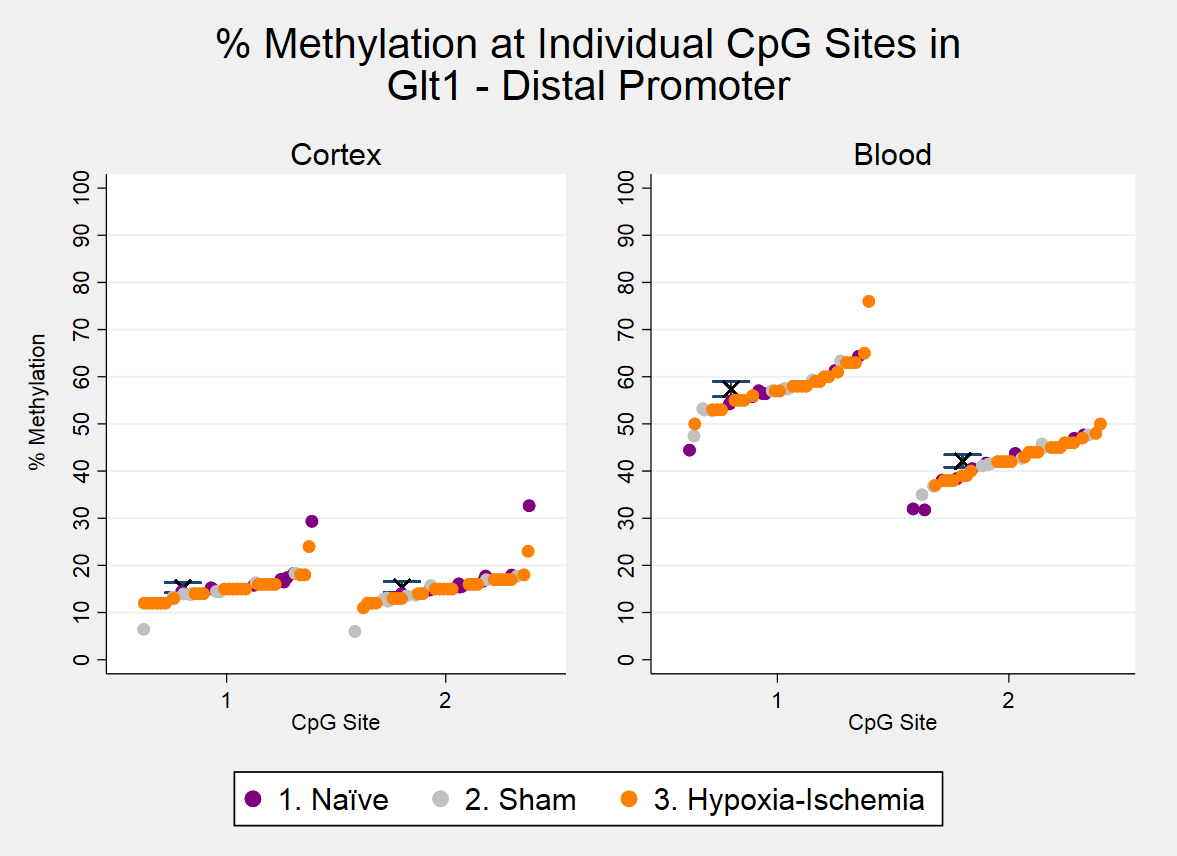
**

Table S25. *Glt1* (distal shore): secondary analyses

For each of the three timepoints for tissue collection (6h, 12h, 24h post-hypoxia): naïve (n=3), sham (n=3), hypoxia-ischemia (n=8). N: naïve; S: sham; HI: hypoxia-ischaemia.

| **Gene** | **Tissue** | **Design of secondary analysis** | **Variable** | **p** |
| --- | --- | --- | --- | --- |
| ***Glt1* (distal promoter)** | ***Cortex*** | *N vs S* | *6h* | 0.500 |
|  |  |  | *12h* | **0.050** |
|  |  |  | *24h* | 0.127 |
|  |  | *N/S vs HI* | *6h* | 0.794 |
|  |  |  | *12h* | 0.474 |
|  |  |  | *24h* | 0.390 |

#### False Discovery Rate

Table S26. DNA methylation in the cortex at 6h: no findings survived Benjamini-Hochberg False Discovery Rate (10%) correction

| **Gene** | **Region** | **Timepoint** | **p-value for Group Effect (Primary Analyses)** | **Rank** | **Benjamini-Hochberg critical value** |
| --- | --- | --- | --- | --- | --- |
| *Proximal* | Cortex | 6h | 0.221 | 1 | 0.033 |
| *Distal* | Cortex | 6h | 0.708 | 2 | 0.067 |
| *CpG Island* | Cortex | 6h | 0.91 | 3 | 0.100 |

Table S27. DNA methylation in the cortex at 12h: no findings survived Benjamini-Hochberg False Discovery Rate (10%) correction

| **Gene** | **Region** | **Timepoint** | **p-value for Group Effect (Primary Analyses)** | **Rank** | **Benjamini-Hochberg critical value** |
| --- | --- | --- | --- | --- | --- |
| *Distal* | Cortex | 12h | 0.101 | 1 | 0.033 |
| *CpG Island* | Cortex | 12h | 0.582 | 2 | 0.067 |
| *Proximal* | Cortex | 12h | 0.995 | 3 | 0.100 |

Table S28. DNA methylation in the cortex at 24h: no findings survived Benjamini-Hochberg False Discovery Rate (10%) correction

| **Gene** | **Region** | **Timepoint** | **p-value for Group Effect (Primary Analyses)** | **Rank** | **Benjamini-Hochberg critical value** |
| --- | --- | --- | --- | --- | --- |
| *Distal* | Cortex | 24h | 0.278 | 1 | 0.033 |
| *CpG Island* | Cortex | 24h | 0.64 | 2 | 0.067 |
| *Proximal* | Cortex | 24h | 0.91 | 3 | 0.100 |

Table S29. DNA methylation in blood at 6h: no findings survived Benjamini-Hochberg False Discovery Rate (10%) correction

| **Gene** | **Region** | **Timepoint** | **p-value for Group Effect (Primary Analyses)** | **Rank** | **Benjamini-Hochberg critical value** |
| --- | --- | --- | --- | --- | --- |
| *Proximal* | Blood | 6h | 0.035 | 1 | 0.033 |
| *CpG Island* | Blood | 6h | 0.304 | 2 | 0.067 |
| *Distal* | Blood | 6h | 0.732 | 3 | 0.100 |

Table S30. DNA methylation in blood at 12h: no findings survived Benjamini-Hochberg False Discovery Rate (10%) correction

| **Gene** | **Region** | **Timepoint** | **p-value for Group Effect (Primary Analyses)** | **Rank** | **Benjamini-Hochberg critical value** |
| --- | --- | --- | --- | --- | --- |
| *CpG Island* | Blood | 12h | 0.04 | 1 | 0.033 |
| *Proximal* | Blood | 12h | 0.169 | 2 | 0.067 |
| *Distal* | Blood | 12h | 0.545 | 3 | 0.100 |

Table S31. DNA methylation in blood at 24h: no findings survived Benjamini-Hochberg False Discovery Rate (10%) correction

| **Gene** | **Region** | **Timepoint** | **p-value for Group Effect (Primary Analyses)** | **Rank** | **Benjamini-Hochberg critical value** |
| --- | --- | --- | --- | --- | --- |
| *Distal* | Blood | 24h | 0.078 | 1 | 0.033 |
| *Proximal* | Blood | 24h | 0.23 | 2 | 0.067 |
| *CpG Island* | Blood | 24h | 0.285 | 3 | 0.100 |
